# Multiscale Modelling of Nanoparticle Distribution in a Realistic Tumour Geometry Following Local Injection

**DOI:** 10.3390/cancers14235729

**Published:** 2022-11-22

**Authors:** George Caddy, Justin Stebbing, Gareth Wakefield, Megan Adair, Xiao Yun Xu

**Affiliations:** 1 Department of Chemical Engineering, Imperial College London, South Kensington Campus, London SW7 2AZ, UK; 2 Department of Surgery and Cancer, Imperial College London, London SW7 2AZ, UK; 3 Xerion Healthcare Ltd., Cherwell Innovation Centre, 77 Heyford Park, Oxfordshire OX25 5HD, UK

**Keywords:** mathematical modelling, radiotherapy, particle transport, tumour

## Abstract

**Simple Summary:**

Nanoparticle radiosensitizers can be used to increase the efficacy of radiotherapy. In this work a multiscale computational model has been developed to assess the distribution of nanoparticles during and after intratumoural injection into a realistic tumour. The aim of the study is to assess how particle surface charge and injection location can affect the distribution of nanoparticles within the tumour, with the optimal result being a uniform concentration across the tumour. This work aims to aid the development of radiosensitizers and guide clinical trials.

**Abstract:**

Radiosensitizers have proven to be an effective method of improving radiotherapy outcomes, with the distribution of particles being a crucial element to delivering optimal treatment outcomes due to the short range of effect of these particles. Here we present a computational model for the transport of nanoparticles within the tumour, whereby the fluid velocity and particle deposition are obtained and used as input into the convection-diffusion equation to calculate the spatio-temporal concentration of the nanoparticles. The effect of particle surface charge and injection locations on the distribution of nanoparticle concentration within the interstitial fluid and deposited onto cell surfaces is assessed. The computational results demonstrate that negatively charged particles can achieve a more uniform distribution throughout the tumour as compared to uncharged or positively charged particles, with particle volume within the fluid being 100% of tumour volume and deposited particle volume 44.5%. In addition, varying the injection location from the end to the middle of the tumour caused a reduction in particle volume of almost 20% for negatively charged particles. In conclusion, radiosensitizing particles should be negatively charged to maximise their spread and penetration within the tumour. Choosing an appropriate injection location can further improve the distribution of these particles.

## 1. Introduction

Radiotherapy is the most commonly used method to treat cancerous tumours, involving the use of high energy particles or waves to cause damage to cancerous cells [1]. Damage is caused through two primary means: DNA targeting, causing strand breaks and therefore no further cell replication; and the generation of highly reactive free radicals. Free radicals are created from the interaction of radiation with oxygen atoms, and will cause significant structural damage to nearby cell [1], a more detailed explanation can be found in our previous work [2].

The particles to be investigated in this study consist of titanium oxide dosed with rare earth metals. X-ray interaction with the particles leads to the generation of free radicals by the splitting of water as well as oxygen. In poorly oxygenated, hypoxic regions of a tumour, the use of nanoparticles is of particular interest. Hypoxic cells are three times more resistant to radiation than well oxygenated cells [3]. In a previous animal study, a xenograft mouse model, using the nanoparticles in conjunction with radiotherapy resulted in a 3.8-fold reduction in tumour regrowth rate versus radiotherapy without the particles [4].

The delivery method chosen is direct intratumoural injection; this has advantages over traditional infusion methods including; reduced systemic toxicity, improved clearance and higher tumour uptake [5]. The damage caused by these radio-sensitizing particles occurs within a nanometer-scale radius, therefore the spatial spread of nanoparticles inside the tumour is an important factor in determining optimal treatment outcomes. Computational modelling of particle transport within a tumour can help to predict the spatial distribution of nanoparticles and making such models physiologically representative is crucial before applying them to real world clinical situations. There has been much previous work in this area including the application of realistic geometry [6,7,8,9], accounting for the effect of tumour vasculature [10,11,12,13,14,15] and for the deformability of the tumour tissue [16,17,18].

In addition, particle deposition onto cell surfaces is an important parameter when modelling the transport of nanoparticles in a tumour as it can affect the nanoparticle distribution. Various methods have been used to account for this effect, including using a constant deposition rate across the tumour [7,19,20] and using correlation equations, first implemented for use in other fields, to calculate the deposition rate [21]. However, both methods have drawbacks, deposition is dependent upon fluid velocity and therefore will be different throughout the tumour, and the correlation equations are not well accepted in the case of charged particles [22].

In this study we extend our previous work to determine the distribution of nanoparticles during and after their injection into a realistic tumour geometry [2]. The particle deposition is calculated using a deposition model rather than previously developed correlation equations. The model is implemented in stages in COMSOL Multiphysics, particle deposition is calculated separately by a micro-model, the macro-model solving for the interstitial fluid velocity and the concentration of nanoparticles is then solved concurrently. A realistic tumour geometry has been reconstructed from micro-CT images of an ex-vivo FaDu tumour. This study investigates the effect of particle surface charge and injection location on the final particle concentration distribution during and after injection.

## 2. Materials and Methods

### 2.1. Mathematical Models

The mathematical model consists of two sub-models of different scales, a macro-model and micro-model.

#### 2.1.1. Micro-Model

The micro-scale model, a particle trajectory tracking model, computes the trajectory of individual particles around a cell. At each time step the forces acting on the particle are evaluated and the corresponding change in position calculated using Newton’s second law of motion [23]:

(1) $$m_{j}\frac{dv_{j}}{dt}=\underset{i}{\sum }F_{i,\;\;j}$$

(2) $$\frac{dr_{j}}{dt}=v_{j}\;\;\;\;\;\;j=1:N_{p}$$

where m is the mass of the particle, v is the velocity of the particle, t is time, $\underset{i}{\sum }F_{i}$ represents the sum of all forces acting on the particle, r is the displacement of the particle and $N_{p}$ is the number of particles.

**Figure 1:**
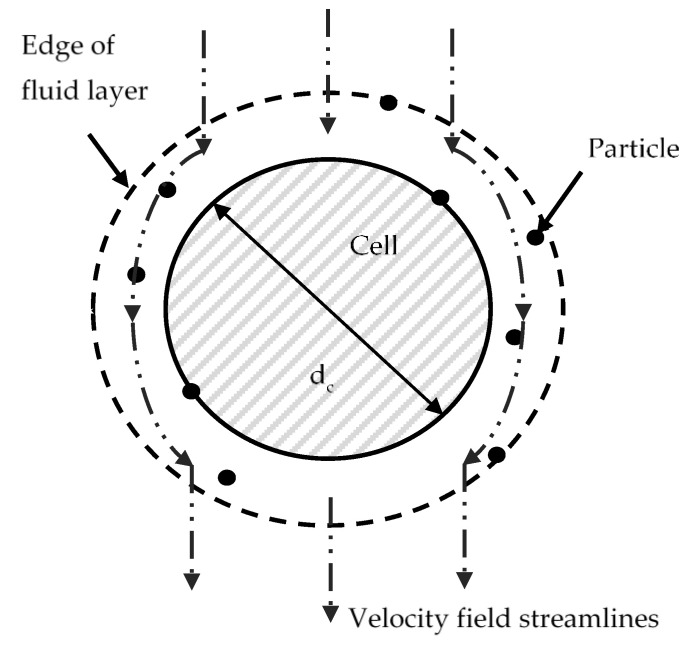
Particle trajectory tracking model geometry, the Happel cell-in-sphere model.

As the tumour is assumed to be a homogeneous porous medium [24], the Happel sphere-in-cell model is used to represent a unit structural cell within the granular porous media. This consists of a solid spherical cell surrounded by a uniform layer of fluid, with the thickness of the fluid layer being dependent upon the porosity of the porous medium, see Figure 1. 

The expression for the fluid velocities in the radial and azimuthal directions can be analytically derived from the stream function of Stoke’s flow, giving [25]:

(3) $$u_{r}=-Ucos\theta \left(\frac{K_{1}}{r^{{*}^{3}}}+\frac{K_{2}}{r^{*}}+K_{3}+K_{4}r^{{*}^{2}}\right)$$

(4) $$u_{\theta }=\frac{1}{2}Usin\theta \left(-\frac{K_{1}}{r^{{*}^{3}}}+\frac{K_{2}}{r^{*}}+2K_{3}+4K_{4}r^{{*}^{2}}\right)$$

(5) $$r^{*}=\frac{2r}{d_{c}}$$

where *U* is the velocity far from the cell, $d_{c}$ is the diameter of the spherical bodies making up the porous medium, r is the particle-cell separation distance, and $K_{1-4}$, are constants given by:

(6) $$K_{1}=\frac{1}{w}\;\;\;\;\;\;K_{2}=\frac{-\left(3+2p^{5}\right)}{w}$$

(7) $$K_{3}=\frac{\left(2+3p^{5}\right)}{w}\;\;\;\;\;\;K_{4}=\frac{-p^{5}}{w}$$

(8) $$w=2-3p+3p^{5}-2p^{6}\;\;\;\;\;\;p={\left(1-\varepsilon \right)}^{\frac{1}{3}}$$

where ε is the porosity of the medium.

There are many forces that can affect the trajectory of a nanoparticle suspended in a fluid [26], but not all will be significant when examining particle movement through a tumour [23]. The forces accounted for in the model are the drag force, lift force, van der Waals force, electrostatic double layer force and Brownian motion.

The drag force is proportional to the difference in velocity between the particle and the fluid [27]:

(9) $$F_{d}=\left(\frac{1}{\tau_{p}}\right)m\left(u-v\right)$$

where $\tau_{p}$ is the particle velocity response time, m is the particle’s mass, u is the fluid velocity and v the particle velocity. For Stokes’s flow, which is applicable in laminar flow conditions, the particle velocity response is given by [22]:

(10) $$\tau_{p}=\frac{\rho_{p}d_{p}^{2}}{18\mu }$$

where μ is the fluid dynamic viscosity, $d_{p}$ is the particle diameter and $\rho_{p}$ particle density. The lift force acts on a nanoparticle moving within a fluid close to a solid surface, the gradient of velocity tends to move the particle in a direction normal to the streamlines of the fluid flow [28]. For this reason, the lift force is assumed to act only in the radial direction. For a non-inertial particle in a laminar flow field close to a surface the following expression for lift velocity was derived [29]:

(11) $$u_{lift}=\frac{55}{144}\frac{\rho_{p}d_{p}u_{f,max}^{2}}{2\mu }{\left(\frac{d_{p}}{2d_{max}}\right)}^{2}\left(1-\frac{d}{d_{max}}\right)\left(1-\frac{73}{22}\frac{d}{d_{max}}\right)$$

where $\rho_{p}$ is particle density, μ is dynamic viscosity of the fluid, d is the particle centre-cell distance, $d=r-d_{c}/2$, $u_{f,max}$ is the maximum fluid velocity, $d_{max}$ is the particle centre-cell distance at maximum velocity. The lift force can then be calculated by [26]:

(12) $$F_{lift}=3\mu \pi d_{p}u_{lift}$$

The van der Waals force accounts for the potential of interactions between the particle and cell surface according to Derjaguin, Landau, Verwey, and Overbeek (DVLO) theory [30]

(13) $$F_{vdW}=\frac{A_{H}d_{p}}{12h^{2}}$$

where $A_{H}$ is the Hamaker constant and is calculated by empirical formulation [31] and h is the surface to surface distance between the particle and the cell:

(14) $$h=r-\left(\frac{d_{c}+d_{p}}{2}\right)$$

The electrostatic double layer force is described by [23]:

(15) $$F_{elec}=-64\pi \varepsilon_{r}\varepsilon_{0}\kappa {\left(\frac{k_{B}T}{zE}\right)}^{2}\left(\frac{d_{p}}{2}\right)\left[tanh\left(\frac{zE\varphi_{1}}{4k_{B}T}\right)tanh\left(\frac{zE\varphi_{2}}{4k_{B}T}\right)\right]e^{-\kappa h}\;$$

where $\varepsilon_{r}$ is the relative dielectric constant, $\varepsilon_{0}$ is vacuum permittivity, $k_{B}$ is the Boltzmann constant, T is temperature, z is the valence of the electrolyte, E is the fundamental charge of an electron, $\varphi_{1}$ is the zeta potential of the particle, $\varphi_{2}$ is the zeta potential of the cell, κ is the Debye-Huckel parameter. This is given by [30]:

(16) $$\kappa =\sqrt{\frac{2N_{A}E^{2}I_{s}}{\varepsilon_{r}\varepsilon_{0}k_{B}T}}$$

where $N_{A}$ is Avogadro’s number and $I_{s}$ is the ionic strength of a solution. Brownian motion describes the random motion of a particle within a fluid arising from collisions between the particle and fluid molecules [32]. The force is defined as [33]:

(17) $$F_{Brown}=\zeta \sqrt{\frac{6\pi k_{B}\mu Td_{p}}{\Delta t\;}}$$

where Δt is the time step of integration, $d_{p}$ is the particle diameter, $k_{B}$ is the Boltzmann constant, T is the absolute temperature, and μ is the fluid dynamic viscosity. ζ is a random value taken from a Gaussian white noise distribution with zero mean and unit variance.

A deposition is counted if the particle makes contact with the cell surface. The collection efficiency, defined as the fraction of the total number of particles simulated for that collide with, and so deposit onto, the cell, can then be found by simulating for a large number of particles [22]. The rate of particle deposition can be calculated through [22]:

(18) $$k_{f}=\frac{3\left(1-\varepsilon \right)}{2\varepsilon d_{c}}\eta_{s}\left|u\right|$$

where $\left|u\right|$ is the magnitude of the local fluid velocity and $\eta_{s}$ is the collection efficiency.

Assumptions used in this model are: particles are assumed to be spherical, particle agglomeration is negligible and particle-particle interactions are neglected.

#### 2.1.2. Macro-Model

The macro-scale model consists of two parts, a nanofluid convection model and a nanoparticle transport model. The nanofluid convection model uses Brinkman’s equations, an extension of Darcy’s law, to calculate for the pressure and flow velocity, the flow velocity is averaged in the representative elementary volume not just the fluid phase [24]. The concentration of the nanoparticles, over space and time, is computed by the nanoparticle transport model. This is described by the convection-diffusion equation with an additional term to describe the deposition of nanoparticles onto the cell surface, which has been shown to significantly affect particle concentration within the fluid [17].

Governing equations for the nanofluid convection and nanoparticle transport models: for more details please refer to our previous work [2].

(19) ∇u=0

Brinkman’s equations:

(20) $$\frac{\rho }{\varepsilon }\left(\frac{\partial u}{\partial t}+\left(u\cdot \nabla \right)\frac{u}{\varepsilon }\right)=-\nabla p+\nabla \cdot \left[\frac{1}{\varepsilon }\left\{\mu \left(\nabla u+{\left(\nabla u\right)}^{T}\right)\;\frac{2}{3}\mu \left(u\cdot \nabla \right)I\right\}\right]-\left(\kappa^{-1}\mu \right)u+F$$

Convection-diffusion-deposition equation:

(21) $$\frac{\partial C}{\partial t}=\nabla \cdot \left(D_{e}\nabla C\right)-\nabla \cdot \left(vC\right)-k_{f}\cdot C$$

### 2.2. Model Geometries

**Figure 2:**
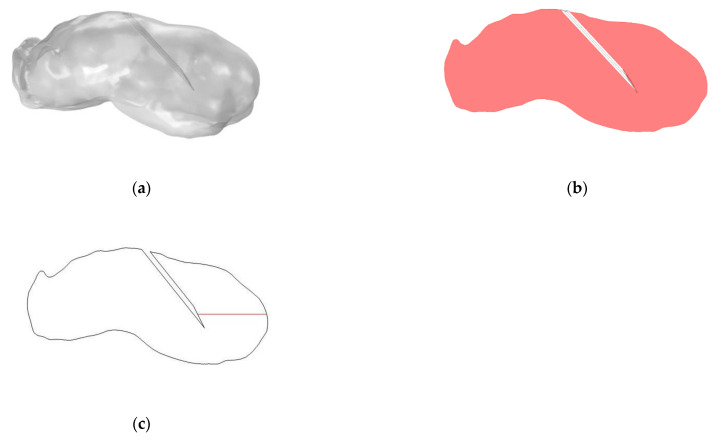
(**a**) Reconstructed FaDu tumour geometry with beveled needle; (**b**) Cut plane through tumour, bisecting the tumour along its long axis; (**c**) Cut line from needle tip to edge of tumour.

There are two geometries used in this model, the particle tracking model simulates flow past an individual cell and is constructed according to the commonly used Happel cell-in-sphere model [25]. For the other two parts of the model the tumour geometry is based on a lab-grown murine tumour, a FaDu cell line is grown in-vivo as a subcutaneous graft in the animal flank. Geometry reconstruction from micro-CT images of this tumour is carried out using Mimics (v20, Materialise, Leuven Belgium). To construct a 3 D geometry from the micro-CT images a threshold pixel intensity value is set to define the region of interest, this is then separated from the unwanted parts of the image and rendered into a 3 D object. This rendering can then be smoothed to fix any reconstruction errors and the final object can be imported to COMSOL as a stereolithography (STL) file. The reconstructed tumour has a volume of approximately 534 mm^3^ and its longest dimension is 15.7 mm. The needle dimensions are that of a 26 s gauge bevelled tip needle [34]. Figure 2 shows the reconstructed tumour geometry and the cut plane used to display the nanoparticle distributions, the cut plane bisects the needle along the longest axis of the tumour. COMSOL Multiphysics (5.5/5.6, COMSOL Inc., Stockholm, Sweden) [35,36] was used to implement all sections of the model. 

### 2.3. Boundary Conditions

For the particle trajectory tracking model, particles are initiated randomly at the edge of the fluid layer along the upstream boundary of the domain. At the cell surface a freeze condition is applied, so any particle that collides with the cell will be deposited. At the downstream boundary of the fluid layer a pass-through condition is applied, so particles that do not collide with the cell will exit the model domain. For the nanofluid convection model a constant velocity is set at the needle tip, while a zero pressure is prescribed at the outer surface of the tumour. For the nanoparticle transport model, the particle concentration is set to be constant at the needle tip, simulating continuous infusion. At the tumour outer surface, a zero-flux condition is applied.

All parameters used in the macro-scale model are given in Table 1.

For the particle trajectory tracking model, the time step must be sufficiently small that the deterministic forces remain constant during the time step but also much larger than the particle momentum relaxation time in order for the assumption of negligible particle inertia to remain valid. The time step used here ranges from 1 × 10^−5^–5 × 10^−8^ s, as the velocity increases the time step must be reduced to fully capture the effect of the random Brownian motion force while also maintaining reasonable computation times. Though the number of particles simulated for can be very large this does not necessitate the introduction of particle-particle interactions or a two-way coupling between the fluid and the particles. This is because the model is not meant to be physically representative, and the number of particles simulated needs to be large enough to ensure repeatability of the collection efficiency value; if the number of collisions is small the collection efficiency could be dramatically varied by only one or two extra collisions. To prevent this the number of particles has been chosen depending upon the particle surface charge and fluid velocity to minimize computation time while maintaining reliable results. However, in reality, the particles are dilute in the fluid and so only a relatively small number will pass by any individual cell, making the effect of particle-particle interactions and particles’ velocity on the fluid velocity negligible. All other simulation parameters used in the micro-scale model are given in Table 2.

### 2.4. Numerical Details

The discretization scheme used for the model was a linear scheme except in the solving of velocity where a quadratic scheme was used, the particle tracing and nanofluid convection models were solved using the generalized minimal residual method (GMRES) and the nanoparticle transport model using the multifrontal massively parallel sparse direct solver (MUMPS). Meshing was done within COMSOL, for the micro-model a finer mesh is applied close to the cell surface, the overall mesh consists of 15,746 triangular elements. The macro-model has a finer mesh implemented around the needle, the mesh consists of triangular surface elements and tetrahedral volume elements, the total mesh consists of 798,416 elements. In the particle tracking geometry, finer mesh was close to the cell surface, and in the realistic tumour geometry close to the needle tip. A mesh independence test was run increasing the number of elements until the less than a 2% change in final concentration results was observed. The micro-model took an average computational time of approximately 1 week for each value of surface charge, the computational time for the macro-model was an average of 2 h across the various surface charges and injection locations.

### 2.5. Particle Diffusivity and Particle Diameter

The uncharged particle diffusion coefficient, $D_{T}$,[38] is determined by the Stokes-Einstein equation as given by [39]:

(22) $$D_{T}=\frac{k_{B}T}{3\pi \mu d_{p}}$$

where $d_{p}$ is the particle diameter, μ is the viscosity of the medium, $k_{B}$ is the Boltzmann constant and T is the absolute temperature. This method for calculating particle diffusion coefficient is only dependent upon particle diameter, however, particle surface charge will also affect diffusivity due to the addition of an electrostatic force between the particles as demonstrated by a recent publication [37]. As such, charged particle diffusivity has been chosen from this publication.

## 3. Results and Discussion

### 3.1. Particle Trajectory Tracking Model

#### Varying Particle Surface Charge

**Figure 3:**
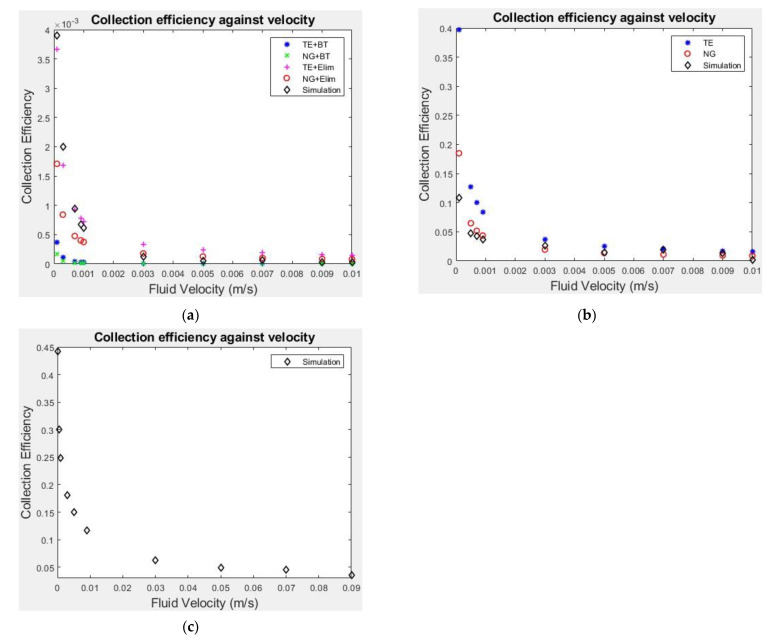
Simulation results for collection efficiency and comparison with results from different correlations: (**a**) for particles with −30 mV surface charge; (**b**) for particles with zero surface charge; (**c**) for particles with +30 mV surface charge. TE: collection efficiency equation from Tufenji and Elimelich [42] attachment efficiency from Bai and Tien [43], NG: collection efficiency equation from Neslon and Ginn [41], Elim: attachment efficiency from Elimelich (Elim) [40].

Figure 3 shows the particle collection efficiency against velocity for three different values of particle surface charge, +30 mV, 0 mV and −30 mV. The results from the particle trajectory tracking model have been compared with those from existing semi-analytical correlation equations for surface charges 0 mV and −30 mV [40,41,42,43]. The chosen semi-analytical correlations were derived by performing regression analysis on previous particle tracking models. They were developed for a variety of fields and each considered a slightly different combination of forces depending upon the particles being studied. For uncharged particles two correlation equations were chosen for comparison: NG [41], represented by red circles in Figure 3b), and TE [42], the blue asterisks. The collection efficiency for charged particles is usually calculated from the combination of two correlation equations, this is because standard correlation equations do not account for the case of unfavourable surface conditions, i.e., both particles having a negative surface charge. To calculate the overall collection efficiency the selected correlation for uncharged particles must be multiplied by an attachment efficiency, the latter can be determined by using correlations BT [43] and Elim [40]. The various combinations of them are as follows: (representation in Figure 3a) TE + BT (blue dots), NG + BT (green crosses), TE + Elim (pink crosses) and NG + Elim (red circles). 

The inclusion of the semi-analytical results helps to evaluate the computational results obtained with our particle tracking model, and to demonstrate the uncertainty in collection efficiency at low velocities. For particles with a negative surface charge this uncertainty can be as large as an order of magnitude. This is because the attachment efficiency correlations for charged particles are based on limited experimental data and so are not widely applicable [22], demonstrating the need for having a tailored deposition model for the specific particles being studied rather than assuming a constant rate or employing correlation equations developed under different conditions [7,19,20,21]. This, therefore, represents a significant change in approach from our previous work where collection efficiency was calculated using the semi-analytical correlation equations [2]. As the correlation equations are not suitable for situations where the particle and the cell have opposing charges, no comparison is made for the positively charged particles. For the uncharged and negatively charged particles, the collection efficiency results for all velocities are within the range predicted by the correlations. The trend of collection efficiency decreasing with increasing velocity is well captured by our particle tracking model and all the selected correlations. Across the range of fluid velocities simulated, the collection efficiency varies by 75% for positively charged particles, by an order of magnitude for uncharged particles and by two orders of magnitude for negatively charged particles. The magnitude of collection efficiency decreases dramatically from positively charged to uncharged to negatively charged particles. Ranging from 0.44 at 1 × 10^−4^ m/s to 0.096 at 1 × 10^−2^ m/s for positively charged particles, 0.11 to 2.1 × 10^−3^ for uncharged particles, and from 3.9 × 10^−3^ to 2.4 × 10^−5^ for negatively charged particles.

The trend of decreasing collection efficiency with velocity seen in all particle surface charge cases is due to the variation of the forces acting on a particle. The motion of particles around the cell is principally determined by the convective force and the random diffusive force caused by collisions of the nanoparticles with surrounding fluid particles, unless the cell-particle distance is extremely small. Therefore, as velocity increases the convective force increases and so the particles will more closely follow the streamlines around the cell. This leads to a reduction in collection efficiency as particles are less able to deviate from the streamlines and collide with the cell. The introduction of a surface charge causes significant variation in collection efficiency, due to the introduction of the electrostatic double layer force in the particle tracking model, which accounts for the force between two charged particles. As cells have a negative surface charge, if the particles are negatively charged this will result in a repulsive electrostatic force and if they are positively charged an attractive electrostatic force. This force is dependent on both the magnitude of the charges and the distance between the particles and only becomes significant when the distance between the particles is very small. For negatively charged particles the force will divert the trajectory of the particles away from the cell and so prevent particle deposition. If the particles are positively charged the force becomes attractive increasing the number of particles colliding with the cell.

### 3.2. Nanoparticle Transport Model

#### 3.2.1. Varying Particle Surface Charge

**Figure 4:**
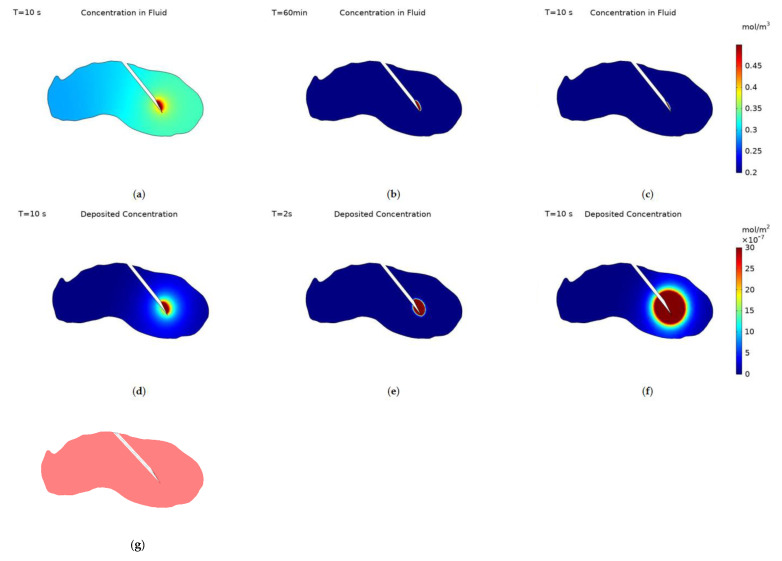
Concentration contours of nanoparticles within the fluid at the end of injection, with surface charge; (**a**) −30 mV, (**b**) 0 V and (**c**) +30 mV, and deposited onto cell surfaces, with surface charge; (**d**) −30 mV, (**e**) 0 and (**f**) +30 mV, (**g**) a schematic of the cut-plane used to plot the results.

Figure 4 displays the nanoparticle concentration within the fluid and deposited onto cell surfaces at the end of injection for the different particle surface charges, −30 mV, 0 and +30 mV. Deposited nanoparticle concentration has the units mol/m^2^ as it is a concentration per unit surface area not per unit volume. The data range for the nanoparticle concentration within the fluid is set to be 0.2–0.5 mol/m^3^ and for the deposited concentration 0–3 × 10^−6^ mol/m^2^ for all three cases, this was to allow for an easy comparison of the effect of surface charge on the spatial concentration of nanoparticles. The spatial profile of nanoparticle concentration within the fluid correlates to the surface charge of the nanoparticles. The negatively charged particles spread much further from the needle tip than uncharged particles, and the spread of uncharged particles is greater than for positively charged particles. All particles concentration profiles follow a similar pattern, a reduced spread of particles behind the needle due to the presence of the needle tip blocking the flow of fluid to the backside of the needle, and the concentration decreasing when moving further away from the injection location. 

The transport of nanoparticles is governed by three transport mechanisms, convection, diffusion, and deposition, and so the variation between the particle surface charge results can be explained within this context. Convection is the same for all three surface charges, as the infusion rate is unchanged, and this drives the growth of the region of high concentration close to the injection location. Particle diffusivity varies dramatically between the charged and uncharged particles, the diffusivity of uncharged particles was calculated from the Stokes-Einstein equation and the diffusivity of the charged particles was experimentally measured. The diffusivities being 7.57 × 10^−12^ m^2^/s for uncharged particles and 1 × 10^−5^ m^2^/s for charged particles. Finally, deposition dictates the number of particles deposited onto the cell surfaces, this is calculated from the collection efficiency results of the particle trajectory tracking model. Deposition is smallest for negatively charged particles, approximately an order of magnitude higher for uncharged particles, and another order of magnitude higher for positively charged particles. As the diffusivity is high and the deposition is low for negatively charged particles there is a significant spread of particles far from the needle tip, for uncharged particles the lack of diffusivity along with the increase in deposition cause the spatial profile of nanoparticle concentration within the fluid to be much reduced. For positively charged particles the increase in deposition is great enough to restrict the concentration profile of nanoparticles within the fluid further despite the increase in diffusivity of the particles. Sometimes one of these transport mechanisms can be left out when simulating for nanoparticle concentration during intra-tumoural injection [12,44,45], however, these results demonstrate how each contributes significantly to the final distribution of nanoparticles and so the importance of including all three. In comparison with our previous work the inclusion of a realistic tumour geometry and measured particle values increase the realism of the model and its usability in conjunction with future clinical trials.

Deposited particle concentration is calculated only from the particle deposition rate and the fluid particle concentration. Therefore, it is expected that the concentration profiles will follow a similar pattern to the concentration of particles within the fluid for each value of surface charge. For negatively charged particles there is a central region with high concentration surrounded by a large region of gently declining concentration to the edge of the close end of the tumour. For uncharged particles the profile is restricted to the immediate vicinity of the needle tip as the diffusivity is low. The size of the central area appears larger than for the concentration within the fluid and this is due to the higher deposition rate of uncharged particles. Although particles can’t penetrate as far into the tumour a far higher fraction will deposit at any point, so the magnitude of deposited concentration will be higher than for negatively charged particles. For positively charged particles there is a large region with high concentration surrounding the needle tip, this is due to a combination of the particles’ high diffusivity and high rate of deposition. The high diffusivity causes a small percentage of particles to penetrate further into the tumour and the high rate of deposition causes almost all of these to deposit onto cell surfaces creating a larger central region of high concentration than for the concentration within the fluid.

**Figure 5:**
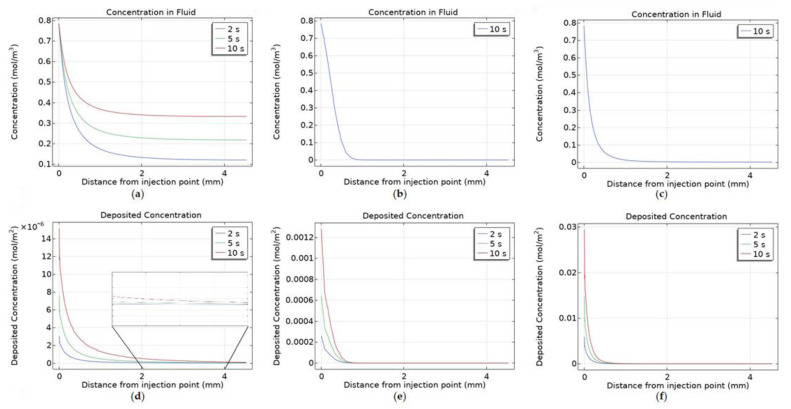
Concentration cut lines of nanoparticles within the fluid at the end of injection, with surface charge; (**a**) −30 mV, (**b**) 0 and (**c**) +30 mV, and deposited onto cell surfaces, with surface charge; (**d**) −30 mV, (**e**) 0 and (**f**) +30 mV.

Figure 5 shows concentration cut lines from the point of injection to the edge of the tumour for the three different surface charges. Highlighting the difference in the spread of the particles both within the fluid and deposited onto cell surfaces. Negatively charged particles within the fluid spread throughout the tumour during the injection with the concentration increasing far from the injection point as the injection progresses, this is a result of the particles’ high diffusivity in conjunction with their low deposition. For uncharged and positively charged particles there is no change in concentration within the fluid during the injection, this is because the transport mechanisms reach a point of equilibrium almost immediately after the start of injection. The influx of particles from convection, the movement of particles from diffusion and the efflux of particles from deposition balance each other. All three types of particles follow the same trend for deposited concentration, with the magnitude of concentration increasing during injection. The maximum value of concentration at the end of injection decreases from positively charged, 0.032 mol/m^2^, to uncharged, 1.40 × 10^−3^ mol/m^2^, to negatively charged particles, 1.6 × 10^−5^ mol/m^2^. The maximum concentration recorded has a negative correlation to the maximum distance from the injection point the particles deposit. This is due to the rate of deposition for particles with different surface charges-for negatively charged particles the rate of deposition is low therefore fewer particles will deposit close to the injection location allowing more to penetrate further into the tumour. In this field the distance from the injection site the particles reach is the main result of concern [12,21,23,45], as most particles injected have a short range of effect. For the particles to significantly aid cancer treatment a uniform spread of particles throughout the tumour is desired, as to leave an area untouched only increases the risk of the treatment not working and the cancer returning.

**Figure 6:**
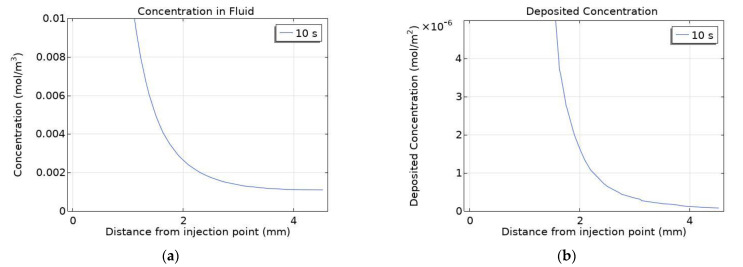
Close-up of concentration cut lines of positively charged nanoparticles: (**a**) within the fluid; (**b**) deposited onto cell surfaces.

Comparing the deposited concentration line and contour for positively charged particles appear to differ significantly, Figure 6 shows a close-up of the previous cut lines for positively charged particles. Demonstrating that the particles within the fluid penetrate further into the tumour than is shown by the overall graph, this is due to the high diffusivity of the particles causing a very small number of particles to be transported far from the injection point. This in turn leads to particles depositing further from the injection point than the overall graph would suggest, as the concentration of particles penetrating further into the tumour is small the concentration of deposited particles will also be small in comparison with the area surrounding the needle tip. However, because the rate of deposition is much higher than the other surface charges the magnitude of deposited concentration will still be similar when comparing for a set data range.

**Figure 7:**
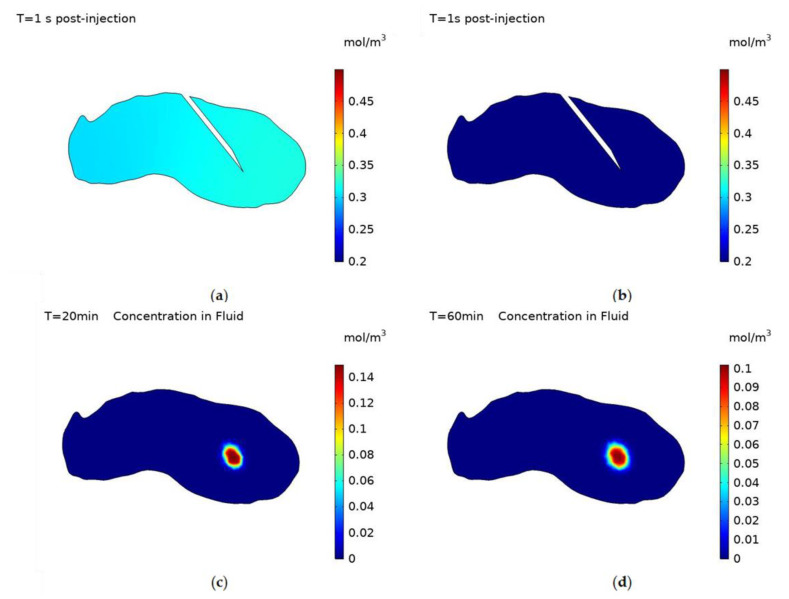
Concentration contours of nanoparticles within the fluid with surface charges; (**a**) −30 mV, (**b**) +30 mV, at 1 s post-injection and for uncharged particles at; (**c**) 20 min and, (**d**) 60 min post-injection.

The concentration within the fluid after the end of injection is shown in Figure 7, both positively and negatively charged particles have high diffusivities and so the concentration reaches equilibrium almost instantly post-injection, with the magnitude being 0.32 mol/m^3^ for negatively charged particles and 1.6 × 10^−3^ mol/m^3^ for positively charged particles. Whereas for uncharged particles the time taken to reach equilibrium is significantly increased due to the particles’ relatively small diffusivity, Figure 7c,d display concentration contours at 20 min and 60 min after the end of injection. Computational constraints meant the simulation was only run for 1-h post-injection and so full equilibrium wasn’t reached for uncharged particles. When simulating for uncharged particles post-injection the needle was removed, as the length of the simulation made it unrealistic for the needle to remain within the tumour. Once equilibrium is reached no further transport of the particles will occur, as convection stops with the end of injection and the rate of deposition is dependent on fluid velocity and so will be zero post-injection.

**Figure 8:**
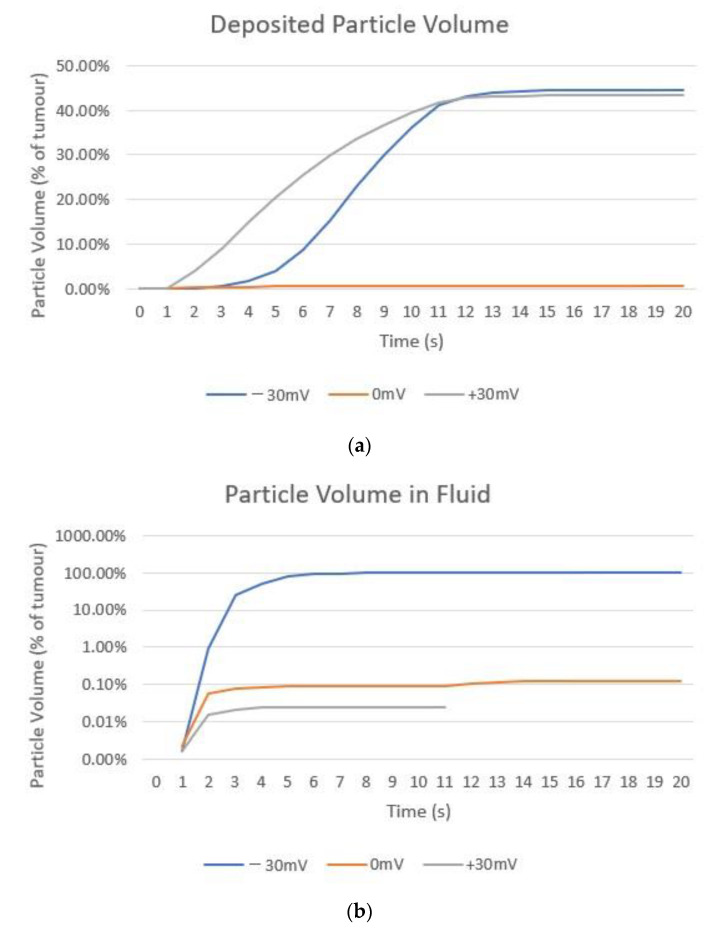
Change in the calculated volume of particles as a percentage of the total tumour volume from the start of injection to 10 s post-injection for all three surface charges: (**a**) deposited onto cell surfaces, threshold 1 × 10^−7^ mol/m^2^; (**b**) within the fluid, threshold 0.1 mol/m^3^.

Particle volumes were calculated to enable a numerical comparison of nanoparticle distribution, this was done by choosing a threshold concentration value to set the particle volume edge. The threshold was set to be 0.1 mol/m^3^ for the volume of particles within the fluid and 1 × 10^−7^ mol/m^3^ for deposited particles. Figure 8 compares the volume of deposited particles and particles within the fluid for all three surface charges during and after the injection. The particle volumes increase during injection and remain constant after injection due to no further particle deposition occurring. The largest deposited particle volume is for negatively charged particles reaching 44.5% of the total tumour volume, then positively charged particles at 43.35% and uncharged particles at only 0.6%. Due to the very large differences in particle volumes within the fluid a logarithmic axis was used for the volume in Figure 8b. The negatively charged particles fill the tumour completely, while uncharged particles occupy 0.1% of the tumour volume and positively charged particles at only 0.01%. Both uncharged and positively charged particle volumes do not increase after 2 s of injection due to the balancing of the transport mechanisms as discussed previously. The shape of the negatively charged particles’ volume is determined by the shape of the tumour, and the positively charged volume ends at 10 s as after this the volume falls to zero and so cannot be plotted on a logarithmic axis. The slight change in volume at the end of injection for uncharged particles is caused by the removal of the needle from the computational domain. A current limitation of the study is the choosing of the concentration thresholds to assess particle volumes, unlike for other nanoparticle treatments [7], at present the therapeutic concentration of the nanoparticles is unknown. Ideally this factor would be known to enable a more accurate picture of useful nanoparticle distribution to be drawn.

#### 3.2.2. Varying Injection Location

**Figure 9:**
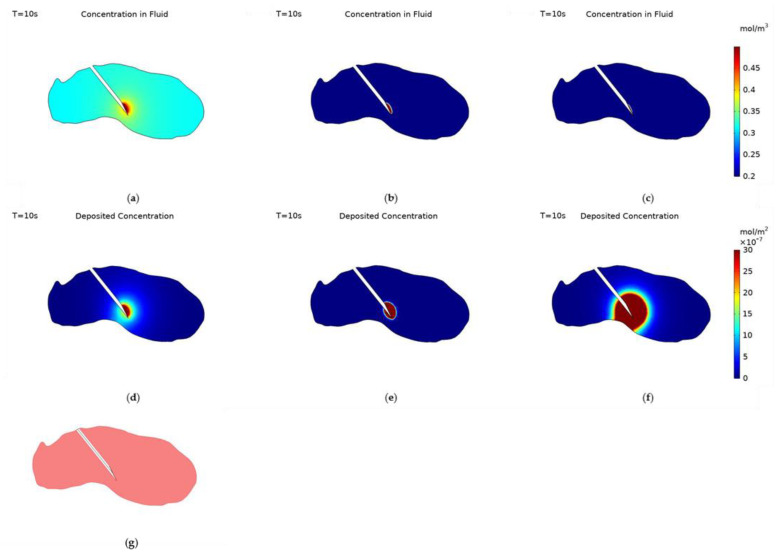
Concentration contours of nanoparticles within the fluid at the end of injection, with surface charge; (**a**) −30 mV, (**b**) 0 and (**c**) +30 mV, and deposited onto cell surfaces, with surface charge; (**d**) −30 mV, (**e**) 0 and (**f**) +30 mV, when the needle is placed centrally in the tumour, (**g**) a schematic of the cut-plane used to plot the results.

The effect of varying injection location on nanoparticle distribution was also investigated. Figure 9 shows the nanoparticle concentration profiles at the end of injection with the needle placed centrally in the tumour for the same three particle surface charges. The concentration profiles follow the same pattern as before, within the fluid the profiles for all surface charges are unchanged from the previous injection location. The concentration profile is restricted to the immediate vicinity of the needle tip for both the positively charged and uncharged cases, for the negatively charged particles the concentration is high in the region surrounding the needle tip and uniformly decreases to a minimum concentration of 0.32 mol/m^3^ throughout the rest of the tumour. Deposited concentration for all particles also follows a similar pattern to the previous injection location, the only difference being that for the positively and negatively charged particles the point of injection is close enough to the edge of the tumour that the profile reaches and surpasses the edge of the domain, the tumour outer surface.

**Figure 10:**
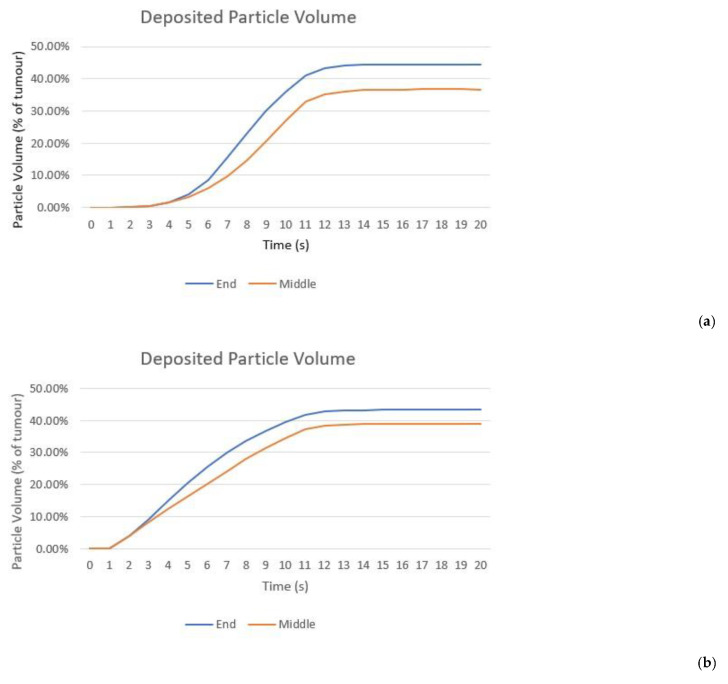
Change in the calculated volume of (**a**) negatively charged; (**b**) positively charged particles as a percentage of total tumour volume deposited onto cell surfaces from the start of injection to 10 s post-injection for two different injection locations, threshold 1 × 10^−7^ mol/m^2^.

Moving the injection location to the centre of the tumour along the long axis has no effect on the particle volume for uncharged particles, the particles cannot move away from the needle tip due to the low diffusivity and the high rate of deposition on cells and so the same results are seen. Figure 10 compares the volume of deposited particles for the two injection locations for the case of negatively and positively charged particles. Showing that the volume for the injection location in the middle of the tumour is substantially smaller than when the needle is placed at the end of the tumour, a decrease of 17.53% for negatively charged particles and 10.14% for positively charged. This is due to spill-over beyond the tumour boundary. The negative effects of this will be two-fold; first a reduction in particle volume within the tumour will reduce the damage caused to cancerous cells and so compromise the efficacy of the radiotherapy. Second, this will cause an increase in damage to the healthy cells surrounding the tumour as the amplification of radiation damage created by the nanoparticles will occur outside of the tumour. Analysing the distribution of nanoparticles from a practical perspective rather than a purely theoretical exercise of obtaining the optimal distribution of nanoparticles is an important step in guiding future clinical trials.

## 4. Conclusions and Future Perspectives

### 4.1. Conclusions

This study updates a multiscale mathematical model to predict nanoparticle concentration during and after direct tumoural injection. The sub-models calculate the fluid velocity, nanoparticle deposition rate and spatio-temporal nanoparticle concentration respectively. The model has several improvements over our previous work [2], an improved deposition model, a realistic geometry and experimentally verified particle parameters. To the authors knowledge this is the first time these improvements have been combined in the same model. The aim of the study was to judge the effect of varying particle surface charge and injection location on the distribution of nanoparticles using a realistic model in order to gain practical insights for future particle design and informing future clinical trials. Particle surface charge has been varied and shown to greatly affect the rate of particle deposition. This, in turn, causes a large variation in the distribution of particles within the tumour, with negatively charged particles giving the most uniform concentration and greatest spread of particles within the tumour. With the volumes of negatively charged particles within the fluid and deposited onto cell surfaces being 100% and 44.5% of the total tumour volume respectively. This is compared to volumes of 0.1% and 0.6% for uncharged particles and 0.01% and 43.35% for positively charged particles. The importance of choosing an optimal injection location is demonstrated by changing the needle placement and comparing simulation results obtained with two different injection locations. The deposited particle volume for negatively charged particles is reduced from 44.5% for the end of tumour injection location to 36.71% for the middle injection location, indicating that an inappropriately selected location could lead to a substantial reduction in the volume of particles remaining within the tumour for negatively charged particles. This could lead to sub-optimal outcomes in the consequent radiotherapy treatment.

### 4.2. Limitations

The current model has several limitations. Firstly, it does not account for the heterogeneous nature of the tumour nor the interactions between the extracellular matrix and nanoparticles. Tumours are known to have a heterogeneous micro-environment with variations in cell density and tissue porosity. Secondly, the vascular network is not included in the model. This could have an effect on the interstitial fluid velocity and doesn’t allow for the heterogeneity of the vascular network and its impact on nanoparticle concentration to be considered [41]. In the future, the model will be improved to incorporate tumour heterogeneity, validated against experimental data and then used to predict injection protocols for future clinical trials.

## Figures and Tables

**Table 1:** Properties and parameters for the nanofluid convection and nanoparticle transport models. (* experimental data).

Parameters and Properties	Values and Source
Injection Amount	0.2 cc (*)
Injection Rate	20 × 10^−4^ L/s (*)
Needle	26 gauge (*)
Nanoparticle Concentration	0.783 mol/m^3^ (*)
Tumour Porosity	0.4 [18]
Tumour Permeability	5 × 10^−13^ m^2^ [18]
Fluid Density	960 kg/m^3^ [18]
Fluid viscosity	3.5 × 10^−3^ kg/(ms) [37]
Nanoparticle diffusivity	7.57 × 10^−12^ m^2^/s (0 mV) 1 × 10^−5^ m^2^/s (+/−30 mV) [37]
Time step	0.1 s

**Table 2:** Additional properties and parameters for the nanoparticle trajectory tracking model. (*experimental data).

Parameters and Properties	Values and Sources
Nanoparticle Density	4230 kg/m^3^
Cell Diameter	15 × 10^−6^ m [18]
Cell Surface Charge	−20 mV [18]
Nanoparticle Diameter	60 × 10^−9^ m (*)
Nanoparticle Surface Charge	−30 mV, 0 mV, 30 mV (*)
Fluid Velocity	1 × 10^−4^–1 × 10^−2^ m/s
Time step	1 × 10^−5^–5 × 10^−8^ s
Number of particles	10,000–1,000,000

## Data Availability

The data presented in this study are available on request from the corresponding author.
